# Hyperphosphatemia Drives Procoagulant Microvesicle Generation in the Rat Partial Nephrectomy Model of CKD

**DOI:** 10.3390/jcm9113534

**Published:** 2020-11-01

**Authors:** Nima Abbasian, Alison H. Goodall, James O. Burton, Debbie Bursnall, Alan Bevington, Nigel J. Brunskill

**Affiliations:** 1 Department of Cardiovascular Sciences, University of Leicester, and Leicester NIHR Cardiovascular Biomedical Research Unit, Leicester LE3 9QP, UK; ahg5@le.ac.uk (A.H.G.); jb343@le.ac.uk (J.O.B.); 57rrd1955@gmail.com (A.B.); njb18@le.ac.uk (N.J.B.); 2 Department of Nephrology, Leicester General Hospital, Leicester LE5 4PW, UK; 3 Division of Biomedical Services, University of Leicester, Leicester LE1 7RH, UK; db79@le.ac.uk

**Keywords:** hyperphosphatemia, chronic kidney disease, cardiovascular disease, endothelial cells, procoagulant MVs

## Abstract

Hyperphosphatemia has been proposed as a cardiovascular risk factor, contributing to long-term vascular calcification in hyperphosphatemic Chronic Kidney Disease (CKD) patients. However, more recent studies have also demonstrated acute effects of inorganic phosphate (Pi) on endothelial cells in vitro, especially generation of pro-coagulant endothelial microvesicles (MV). Hitherto, such direct effects of hyperphosphatemia have not been reported in vivo. Thirty-six male Sprague-Dawley rats were randomly allocated to three experimental groups: (1) CKD induced by partial nephrectomy receiving high (1.2%) dietary phosphorus; (2) CKD receiving low (0.2%) dietary phosphorus; and (3) sham-operated controls receiving 1.2% phosphorus. After 14 days the animals were sacrificed and plasma MVs counted by nanoparticle tracking analysis. MVs isolated by centrifugation were assayed for pro-coagulant activity by calibrated automated thrombography, and relative content of endothelium-derived MVs was assessed by anti-CD144 immunoblotting. When compared with sham controls, high phosphorus CKD rats were shown to be hyperphosphatemic (4.11 ± 0.23 versus 2.41 ± 0.22 mM Pi, *p* < 0.0001) with elevated total plasma MVs (2.24 ± 0.37 versus 1.31 ± 0.24 × 10^8^ per ml, *p* < 0.01), showing increased CD144 expression (145 ± 25% of control value, *p* < 0.0001), and enhanced procoagulant activity (18.06 ± 1.75 versus 4.99 ± 1.77 nM peak thrombin, *p* < 0.0001). These effects were abolished in the low phosphorus CKD group. In this rat model, hyperphosphatemia (or a Pi-dependent hormonal response derived from it) is sufficient to induce a marked increase in circulating pro-coagulant MVs, demonstrating an important link between hyperphosphatemia and thrombotic risk in CKD.

## 1. Introduction

Renal function inversely correlates with cardiovascular mortality in humans [1]. Elevation of plasma inorganic phosphate (Pi) (hyperphosphatemia) in Chronic Kidney Disease (CKD) is thought to be an important contributor to this, partly because of Pi’s effect on calcium deposition, resulting in vascular calcification [2,3,4,5,6,7]. However, effects of elevated soluble Pi, apparently independent of calcium, have also been demonstrated in vitro and in vivo, for example, direct effects on parathyroid [8] and endothelial cell (EC) dysfunction [9,10,11,12,13]. Endothelial effects are of particular interest because CKD patients have been shown to have elevated circulating concentrations of pro-coagulant microvesicles (MVs) derived from endothelial cells, leading to a prothrombotic state, which may contribute to acute occlusive events [14,15,16]. MVs are submicron diameter vesicles shed from several cell types, notably platelets and vascular endothelial cells, following apoptosis or cellular activation [9,10,17,18]. They occur in plasma of healthy subjects, but their abundance, both from platelets and ECs, has been shown to increase in CKD patients [14]. We previously showed that applying elevated extracellular Pi concentration to cultured ECs is sufficient to trigger their rapid release [9] through direct inhibition by Pi of the phosphoprotein phosphatase PP2A in ECs, culminating in cytoskeleton disruption and MV generation [10], an effect which may explain the pro-coagulant endothelial MVs previously reported in CKD patients [14]. However, a direct pro-coagulant effect of hyperphosphatemia has not been reported in vivo.

We therefore hypothesised that hyperphosphatemia in CKD in vivo is sufficient to trigger an increase in circulating pro-coagulant endothelial MVs, and that correction of hyperphosphatemia by feeding a low phosphorus diet could correct this. The study was performed using a rat partial nephrectomy model of CKD because the dietary phosphorus loads of 1.2% by weight (needed to induce stable hyperphosphatemia) and of 0.2% by weight (needed to correct hyperphosphatemia) have previously been well defined in this model [19].

## 2. Materials and Methods

### 2.1. Rat Partial Nephrectomy Model of CKD

**Scheme 1:**
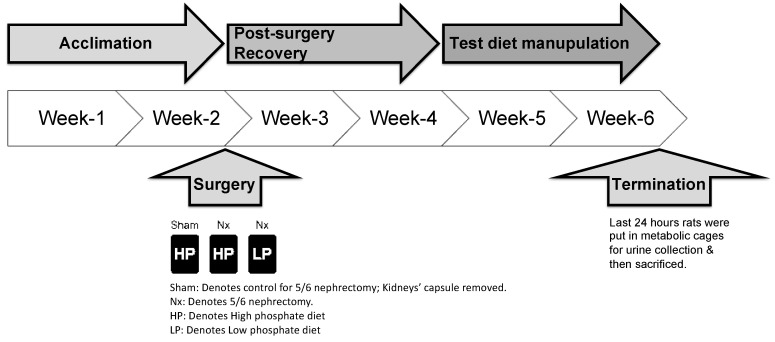
Schematic overview of in vivo study design.

Scheme 1 shows a schematic overview of the study design. All surgical and experimental procedures were performed subject to project licence reference PPL P444C43C0 under the Animals (Scientific Procedures) Act (United Kingdom, 1986), and were approved by the University of Leicester Animal Welfare and Ethical Review Body. Male Sprague-Dawley (SD) rats were purchased from Charles River UK at ~140–160 g. Rats (12 in each study group) were acclimatised on normal rat diet containing 0.56% phosphorus (Test Diet Limited, BCM IPS Ltd., London, UK) for 14 days before commencing surgery. A one-stage partial nephrectomy (designated “Nx” throughout this study) was performed on 24 of the resulting ~225–250 g rats under general anaesthesia as described previously [20] but a dorsal incision was used in place of abdominal access to the kidneys. Briefly, rats were anaesthetised (3.5% isoflurane in oxygen delivered at 3 litres per min), accompanied by sub-cutaneous administration of Rimadyl (Carpofen) 4 mg/kg body weight) for post-operative analgesia. Anaesthesia was maintained during surgery using 2.5% isoflurane in oxygen at 1 litre/min. The foot withdrawal reflex was tested to confirm anaesthesia. The whole of the right kidney and approximately 0.4 g of the left kidney (mainly from the cortex) were excised. The remaining 12 rats (designated “Sham” throughout this study) were anesthetised and subjected to exposure and decapsulation of both kidneys, but no excision of tissue was performed.

After a 14 day recovery period from the surgery, in which all rats were fed on the 0.56% phosphorus diet, the 24 Nx rats were randomly allocated to experimental diets: 12 receiving high a (1.2%) phosphorus diet for 14 days (referred to as “NxH” throughout this study), and 12 receiving 14 days of low (0.2%) phosphorus diet (referred to as “NxL”). Throughout this 14-day experimental period, the Sham rats were pair fed with the NxH and NxL rats, with water ad libitum. (The NxH and NxL rats received the same weight of diet but diets differed in the phosphorus content supplied). For the last 24 h rats were housed separately in metabolic cages to allow collection of urine. Thereafter, animals were sacrificed under general anaesthesia by exsanguination via cardiac puncture. Blood was collected for biochemical and MV analysis. For isolation of MVs, blood was collected into citrated tubes and MVs were separated by differential centrifugation, as previously described [9,14].

### 2.2. Blood and Urine Biochemistry

Levels of creatinine (Cr), total calcium, inorganic phosphate (Pi), and urea were measured with commercially available kits (Universal Biologicals Ltd.). Renal clearance of creatinine (expressed as mL/min/kg body weight) was calculated using the following formula:

((Cu/Cp) × Vu)/BW

where Cu is the concentration of creatinine in urine, Cp is the concentration of creatinine in plasma, Vu is the average rate of urine production (in mL per minute) during the urine collection period, and BW is the body weight in kg.

### 2.3. Nanoparticle Tracking Analysis (NTA)

The number and size of the particles in the isolated MV samples was analysed by nanoparticle tracking analysis (NTA) using a NanoSight LM10 analyser with NTA software v2.2 (NanoSight Ltd., Amesbury, UK) and 90 s video capture as previously described [9,14].

### 2.4. Thrombin Generation Assay (TGA) Using Calibrated Automated Thrombography

The ability of isolated MVs, reconstituted in EV-free plasma pooled from 20 healthy donors, to enhance thrombin generation was determined as previously described [21] using 1 × 10^6^ MVs per thrombin generation assay (TGA) reaction (counted by NTA as described above) by calibrated automated thrombography with Platelet-Rich Plasma Reagent (Diagnostica Stago) containing 1 pM tissue factor.

### 2.5. Immunoblotting

Isolated plasma MVs lysed in cell lysis buffer with 49.5 mM Tris, pH 8; 150 mM NaCl; 1% Nonidet P-40; and 1% phenylmethylsulfonyl fluoride were subjected to SDS-PAGE followed by immunoblotting. Immunoblotting was performed on nitrocellulose membranes (Amersham) followed by probing with primary mouse monoclonal antibody against CD144 (Insight Biotechnology). Polyclonal rabbit anti-mouse immunoglobulins (HRP conjugated) (DakoCytomation) were used as the secondary antibody and HRP-labelled protein was detected by chemiluminescence (ECL-Amersham). Band intensities for CD144 were quantified by ImageJ and data are presented as fold-changes compared to the sham-operated group, as the ratio of the intensity for the protein of interest (CD144)/total proteins. Cumulative intensity of total proteins detected on BioRad Mini Protean TGX stain-free gels was quantified using the stain-free gel detection facility on a BioRad ChemiDocTM Touch imaging system prior to transfer of the proteins onto the nitrocellulose membranes.

### 2.6. Statistical Analyses

Data are presented as bar graphs together with the mean ± SEM and were analysed using GraphPad Prism 8.0. Sample size denotes the number of rats in each study group. Differences among groups were analysed by one-way ANOVA, followed by Tukey’s multiple comparisons test, for normally-distributed data, and Dunn’s multiple comparisons test, for non-normally distributed data. *p* values < 0.05 were considered statistically significant. 

## 3. Results

**Figure 1:**
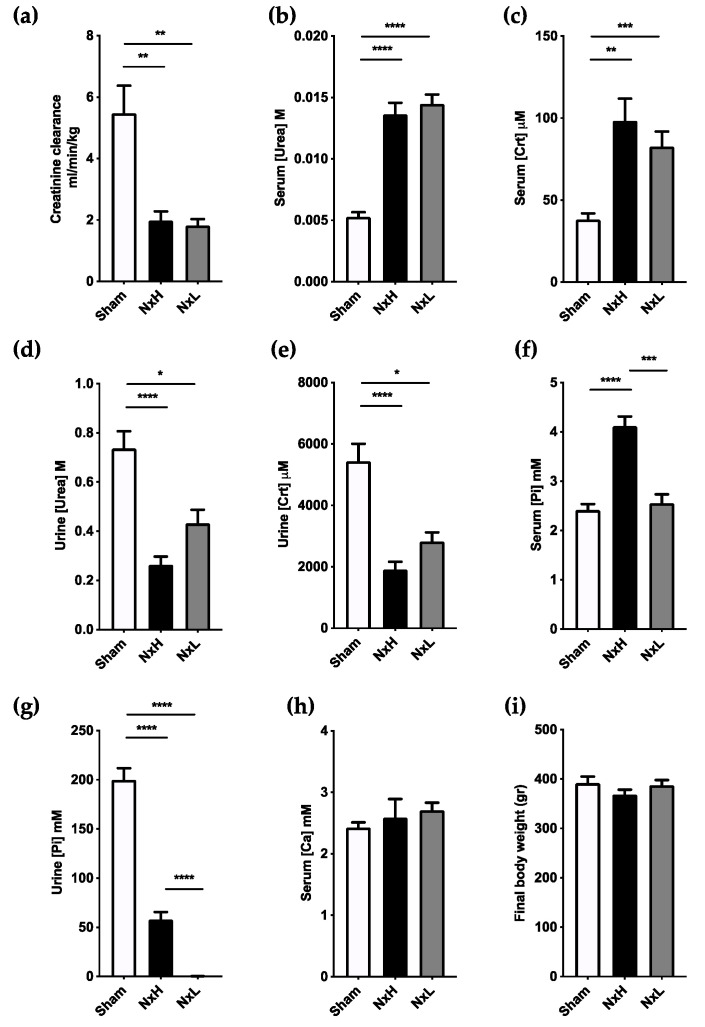
Development of renal failure in Chronic Kidney Disease (CKD) rats. (**a**); Creatinine clearance, (**b**); Serum (Urea), (**c)**; Serum Creatinine (Crt), (**d**); Urine (Urea), (**e**); Urine (Crt), (**f**); Serum inorganic phosphate (Pi), (**g**); Urine (Pi), (**h**); Serum total Calcium (Ca), (**i**); Final body weight. Sham denotes control rats subjected to bilateral kidney decapsulation and receiving a high (1.2%) phosphorus diet for 14 days after two weeks’ recovery from the surgery. NxH denotes partially nephrectomised rats receiving a high (1.2%) phosphorus diet for 14 days after two weeks’ recovery from the surgery. NxL denotes partially nephrectomised rats receiving a low (0.2%) phosphorus diet for 14 days after two weeks’ recovery from the surgery. *n* = 12; (12 rats in each study group), * *p* < 0.05, ** *p* < 0.005, *** *p* < 0.0005,**** *p* < 0.0001.

At 28 days after surgery (Scheme 1), the rat partial nephrectomy model of CKD in NxH and NxL rats showed significantly reduced renal function (assessed from creatinine clearance) (Figure 1a), and elevated serum concentrations of urea and creatinine (Figure 1b,c), when compared with that of Sham-operated control rats. Animals in all three experimental groups were successfully matched for food consumption (average cumulative food consumed over 14 days of receiving test diet; 246.12 g vs. 230.4 g vs. 257.8 g for Sham, NxH, and NxL respectively) and showed no significant difference in final body weight (Figure 1i).

As expected, renal insufficiency in NxH rats on a high phosphorus diet resulted in phosphate retention [22] shown by increased serum Pi concentration (Figure 1f) and decreased urinary Pi excretion (Figure 1g) compared with that of Sham-operated rats receiving the same dietary phosphorus intake. Feeding a low phosphorus diet to CKD rats in group NxL corrected hyperphosphatemia (Figure 1f) and abolished urinary excretion of Pi (Figure 1g). Serum calcium concentration was unaffected across the three study groups (Figure 1h).

**Figure 2:**
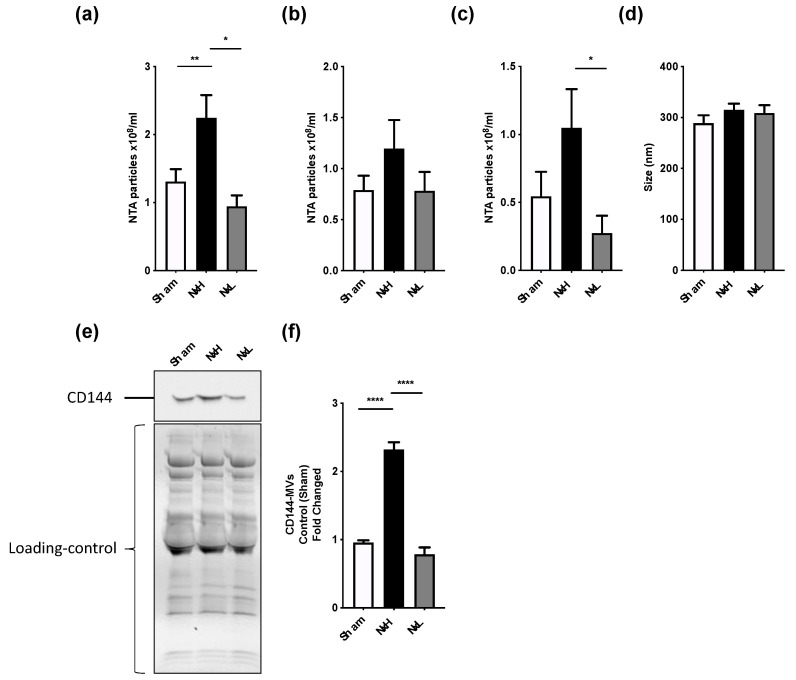
Hyperphosphatemia induces release of endothelial microvesicles (MVs). (**a**–**d**) Count and size range of MVs as measured by nanoparticle tracking analysis (NTA). (**a**); Total MVs, (**b**); MVs sized between 100–1000 nm, (**c**); 10–100 nm diameter particles, (**d**); particle size. (**e**,**f**) Representative Western blot results and densitometry analysis, respectively, showing the presence of CD144+ endothelium-derived MVs within isolated plasma MVs. Sham denotes control rats subjected to bilateral kidney decapsulation and receiving a high (1.2%) phosphorus diet for 14 days after two weeks’ recovery from the surgery. NxH denotes partially nephrectomised rats receiving a high (1.2%) phosphorus diet for 14 days after two weeks’ recovery from the surgery. NxL denotes partially nephrectomised rats receiving a low (0.2%) phosphorus diet for 14 days after two weeks’ recovery from the surgery. *n* = 12; (12 rats in each study group), * *p* < 0.05, ** *p* < 0.005, **** *p* < 0.0001.

As an increase in extracellular Pi concentration comparable with that in the NxH group (Figure 1f) had previously been shown to induce release of MVs from cultured ECs [9], the corresponding effect on MVs was investigated here in the rats’ plasma (Figure 2a–c). As predicted, the MV concentration was significantly higher in the hyperphosphatemic NxH group compared with the Sham operated group, reaching statistical significance for the total MV pool (Figure 2a) and for the 10–100 nm fraction of MVs (Figure 2c). A similar upward trend was observed for the 100–1000 nm microparticle fraction (Figure 2b) but fell short of statistical significance (NxH versus Sham, *p* = 0.351, see Discussion). Correction of hyperphosphatemia (Figure 1f) by feeding the low phosphorus diet in the NxL rats abolished the increase in the MV count (Figure 2a–c). The average particle diameter showed no significant difference across the three experimental groups (Figure 2d).

A significant contribution from endothelial vesicles to the increase in plasma MVs in the NxH rats was confirmed by immunoblotting for the endothelial marker CD144 performed on lysates from isolated MVs (Figure 2e,f). As for the MV count in Figure 2a,c, the increase in the CD144 signal observed in the NxH group was abolished in the low phosphorus NxL group (Figure 2e,f).

**Figure 3:**
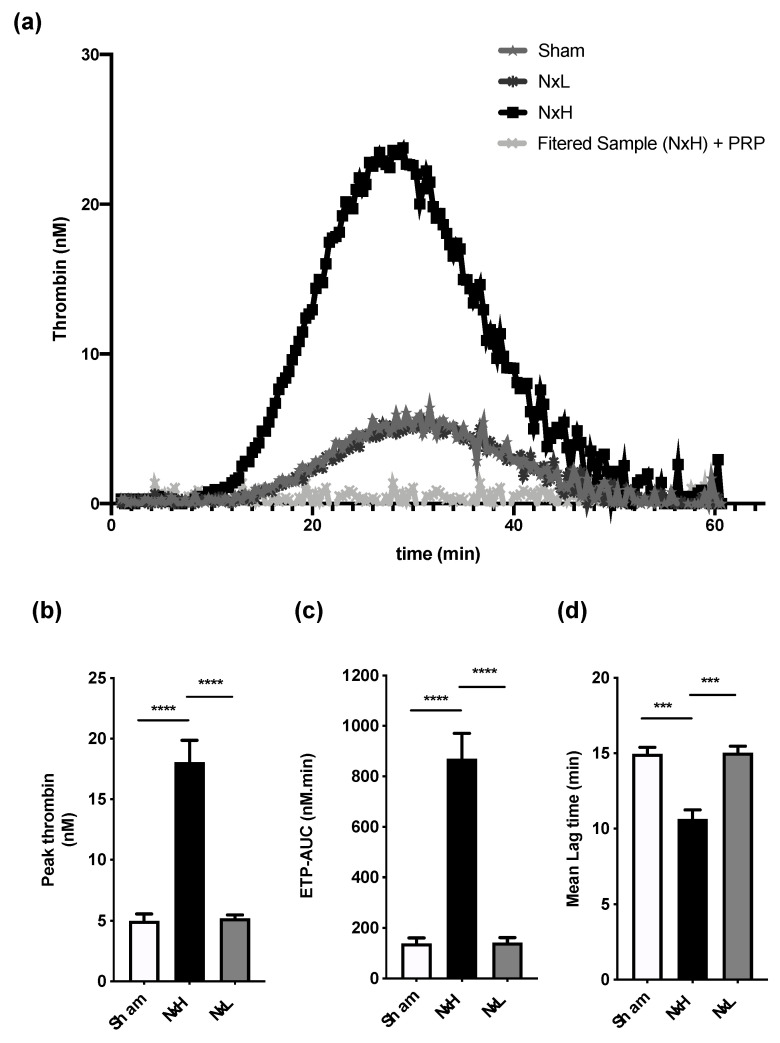
Calibrated automated thrombography showing the prothrombotic effects of MVs in rats’ plasma. (**a**) Representative thrombin generation curves form each subject group (**b**) Peak thrombin, (**c**) Endogenous thrombin potential-area under the curve (ETP-AUC), and (**d**) Lag-time. Sham denotes control rats subjected to bilateral kidney decapsulation and receiving a high (1.2%) phosphorus diet for 14 days after two weeks’ recovery from the surgery. NxH denotes partially nephrectomised rats receiving a high (1.2%) phosphorus diet for 14 days after two weeks’ recovery from the surgery. NxL denotes partially nephrectomised rats receiving a low (0.2%) phosphorus diet for 14 days after two weeks’ recovery from the surgery. *n* = 12; (12 rats in each study group), *** *p* < 0.0005, **** *p* < 0.0001.

To confirm that the Pi-induced increase in MVs exerted a net pro-coagulant effect (as previously reported for Pi-induced MVs generated from cultured endothelial cells [9]), MVs isolated by centrifugation were assayed for pro-coagulant activity by calibrated automated thrombography (Figure 3). Both the peak thrombin concentration (Figure 3b) and endogenous thrombin potential-area under the curve (ETP-AUC) (Figure 3c) were significantly elevated in the MVs derived from the NxH rats when compared with that of Sham-operated controls. Onset of coagulation was also accelerated, shown by a reduced lag time (Figure 3d). All of these effects, which are indicative of an increased presence of procoagulant MVs, were abolished when hyperphosphatemia was corrected by feeding a low phosphorus diet in NxL rats (Figure 3b–d) and also when NxH MVs were removed by filtration (Figure 3a).

## 4. Discussion

### 4.1. Contribution of Hyperphosphatemia to Pro-Coagulant MV Load in CKD

It has previously been shown that the concentration of cell-derived MVs circulating in plasma is significantly elevated in patients with CKD [14,15,23,24,25], that these particles are principally of endothelial, platelet, and monocyte origin, that they exert a potent pro-coagulant effect [14], and that in vitro elevated extracellular Pi concentration triggers rapid release of MVs from cultured ECs [9]. The data in the present study now provide a key advance in this field, supporting the hypothesis that in CKD it is hyperphosphatemia that triggers release of pro-coagulant MVs into circulation. It was also shown that hyperphosphatemia increased the circulating concentration of endothelial MVs (although to what extent these endothelial MVs account for the increased pro-coagulant effect remains to be shown in future work). As feeding a low phosphorus diet to CKD rats brought serum Pi concentration down to the level observed in rats with intact kidneys, and restored MV numbers and coagulation state to control level, these effects of CKD were almost entirely attributable to Pi, possibly through a direct effect of Pi ions on endothelial cells as previously described in vitro [9]. It might be argued that the effects of high versus low phosphorus diet observed in the NxH versus NxL CKD rats in Figure 1, Figure 2 and Figure 3 arose instead from some indirect effect of dietary phosphorus (e.g., a hormonal signal from the gastro-intestinal tract to endothelium). However, feeding the same high phosphorus diet to sham control rats with intact kidneys, which yielded no hyperphosphatemia (Figure 1f), exerted no such effects on MVs, suggesting that the effects reported here are an action of elevated plasma Pi on endothelium. It should be emphasized, however, that the possibility of an indirect hormonal effect of hyperphosphatemia on endothelium (for example, a Pi-induced change in the circulating concentration of phosphatonins, such as parathyroid hormone or FGF23, which may then act on the endothelial cells) has not been ruled out, and studies of such factors would be an interesting subject for future research in this field.

### 4.2. Role of Endothelial Versus Platelet-Derived MVs

In this study hyperphosphatemia induced a clear increase in the total MV pool and the population in the size range 10–100 nm. Furthermore immunoblotting for endothelium-specific membrane protein CD144 in MV preparations from the NxH group of hyperphosphatemic CKD rats confirmed the increased abundance of endothelial MVs (Figure 2e,f). However, unlike our earlier in vitro study in cultured endothelial cells [9], in which 2.5 mM Pi triggered release of endothelial MVs in the microparticle size range of 100–1000 nm diameter, here, an apparent increase was seen in this range but failed to reach statistical significance (Figure 2b). It is important to emphasise, however, that a key difference between the earlier in vitro study and the present in vivo study is that in plasma in vivo the majority (typically 60%) of the particles detected in the ~100–1000 nm microparticle size range are of platelet origin [26], thus partly obscuring the hyperphosphatemia-induced rise in endothelial microparticles that was anticipated in this size range. It is unlikely that platelet microparticle numbers will increase in response to hyperphosphatemia. MV release from cultured endothelial cells in response to high extracellular Pi concentration has been shown to be triggered by the resulting increase in intracellular Pi concentration [9]. In contrast, in human platelets the steady-state intracellular Pi concentration is unaffected by hyperphosphatemia imposed in vivo [27]. Consistent with this observation, applying a 2.5 mM extracellular Pi load to human platelets for 90 min in our laboratory had no detectable effect on release of platelet MVs.

### 4.3. Clinical Implications

Evidence presented here (Figure 3) suggests that hyperphosphatemia contributes to increased thrombotic risk by triggering release of pro-coagulant endothelial MVs, consistent with previous evidence of pro-coagulant MVs in circulation in dialysis patients [14]. Venous thromboembolism (VTE), including deep vein thrombosis and pulmonary embolism (PE), are associated with CKD in patients [28], and decreasing eGFR has been shown to be an independent risk factor for VTE [28]. Furthermore, progression of CKD is associated with progression of atherosclerosis [29] and increased risk of blood clotting at the site of plug rupture.

The present study was not designed to investigate the time course of the effect of hyperphosphatemia on MV release in vivo. The effect was studied here after 2 weeks of dietary phosphorus intervention to ensure that a clear stable hyperphosphatemia developed in the partially nephrectomised rats [19]. However, if direct action of Pi on endothelial cells is a significant contributor to the effects reported here, onset of thrombotic risk in response to hyperphosphatemia is likely to be more rapid than 2 weeks. Work from our laboratory [9] and others [11,12,30] indicates that (at least in vitro) the response of ECs to elevated extracellular Pi concentration is rapid, and significant release of pro-coagulant MVs in vitro in response to 2.5 mM Pi occurs in as little as 90 min. This is a radically different time scale from the long-term exposure to high Pi required to induce vascular calcification. This implies that, in addition to the well-documented problems involved in managing the ubiquitous presence of phosphorus in diets [31,32,33,34,35], even relatively brief post-prandial “spikes” of hyperphosphatemia (which have been well documented in CKD [36,37,38]) may pose a significant risk.

## 5. Conclusions

In conclusion, this study is the first demonstration in a CKD model in vivo that hyperphosphatemia (or a Pi-dependent hormonal response derived from it) drives accumulation of pro-coagulant MVs, and hence identifies Pi as a clearly defined biochemical target for future measures to reduce thrombotic disease in CKD.
